# Local Perceptions, Cultural Beliefs, Practices and Changing Perspectives of Handling Infant Feces: A Case Study in a Rural Geita District, North-Western Tanzania

**DOI:** 10.3390/ijerph17093084

**Published:** 2020-04-29

**Authors:** Joy J. Chebet, Aminata Kilungo, Halimatou Alaofè, Hamisi Malebo, Shaaban Katani, Mark Nichter

**Affiliations:** 1 Department of Health Promotion Sciences, Mel and Enid Zuckerman College of Public Health, The University of Arizona, Tucson, AZ 85724, USA; 2 Department of Environmental Health Sciences, Mel and Enid Zuckerman College of Public Health, The University of Arizona, Tucson, AZ 85724, USA; 3 National Institute for Medical Research, 11101 Dar es Salaam, Tanzania; 4 Department of Anthropology, University of Arizona, Tucson, AZ 85724, USA

**Keywords:** infant feces disposal, child feces management, diarrhea transmission, perceptions, culturally relevant interventions, Geita Region, Tanzania

## Abstract

We report on the management of infant feces in a rural village in Geita region, Tanzania. Findings discussed here emerged incidentally from a qualitative study aimed at investigating vulnerability and resilience to health challenges in rural settings. Data was gathered through semi-structured focus group discussions (FDGs) with women (*n* = 4; 32 participants), men (*n* = 2; 16 participants), and community leaders (*n* = 1; 8 participants). All FDGs were audio recorded, transcribed verbatim and thematically analyzed using Atlas.ti. Respondents reported feces of a child under the age of six months were considered pure compared to those of older children. Infant feces were seen as transitioning to harmful at the point when the child began to eat solid food, resulting in their stool visually changing in appearance. Caregivers reportedly used soft implements to handle infant feces due to the belief that tools with hard surfaces would physically harm the child. Infant feces were disposed in environments around the house due to the belief that disposal in latrines would prevent developmental milestones and result in other perceived negative health outcomes for the child. Changing views expressed by participants suggest a window of opportunity to implement evidence-based and culturally relevant interventions to encourage the safe disposal of infant feces.

## 1. Introduction

Globally, water, sanitation, and hygiene (WaSH)-related diseases remain one of the leading causes of death among children under the age of five [1]. Of the approximate 1.6 million annual diarrheal cases in this population worldwide, an estimated 446,000 (26%) result in death from complications associated with the infection [1]. Overwhelmingly, these deaths occur in low-resource settings in sub-Saharan Africa and South East Asia, and are largely attributable to unsafe water and inadequate sanitation [1]. Furthermore, repeated gastrointestinal infections result in additional downstream effects such as environmental enteropathy (EE), whose impacts on health are more difficult to quantify [2,3]. EE, a subclinical condition, results in the blunting of intestinal villi and has been linked to malabsorption of nutrients, malnutrition, impaired cognitive function, and vaccine failure in children [4,5,6]. Taken together, exposure to fecal contamination—through water or the environment—and subsequent sequalae of infection contributes significantly to global mortality and morbidity across all age groups, but display more pronounced consequences in children under five years of age [7,8].

Proper disposal of human excreta mitigates transmission of pathogens through fecal-oral routes [9,10]. Young children tend to defecate close to the home, where fecal matter and associated pathogens can contaminate the environment and more easily be transmitted to other children and adults [11,12]. The handling and disposal of child feces is especially important since the stool of children have been shown to contain more pathogens than that of adults [9]. Furthermore, children spend a lot of time on the ground exploring the physical environment, and may engage in actions such as putting objects, sand, and other materials in their mouths, which may expose them to these pathogens [13,14,15]. This, in addition to their underdeveloped immune systems place young children at greater risk of enteric infections [3].

Risky child feces management behaviors have been associated with increased incidence of diarrhea [16]. A meta-analysis of 6 studies carried out in South East Asia showed these behaviors increased the risk of diarrheal diseases in under-five children by 23% (RR: 1.23, 95% CI: 1.15–1.32) [16]. Additionally, a pooled analysis of six case-control studies showed that disposal of a child’s feces into a latrine decreases the odds of diarrhea by about 25% in children under five years of age (OR: 0.73, 95% CI 0.62–0.85) [17]. The World Health Organization (WHO) and United Nations Children’s Fund (UNICEF) joint monitoring program (JMP) therefore advocates for safe child feces disposal. This is defined as a child using an improved toilet, and/or a caregiver discarding a child’s stool into an improved toilet, where an improved toilet is one that hygienically separates feces from human contact [18]. Therefore, burying feces, leaving it in the open, or throwing it in the garbage are not considered safe stool disposal practices [18]. 

According to the 2015/16 Tanzanian Demographic and Health Survey (DHS), 65.7% of children in the Geita region aged five and below had their stool disposed of safely into an improved sanitation facility or buried the last time they defecated [19]. This is compared to a national average of 71.9% (83.0% urban vs. 67.7% rural) [19]. Moreover, the safe disposal of child feces decreased with increased age, with 44.1% of Tanzanian children under the age of six months having their stool safely disposed of [19]. This is in comparison to 72.4% of children aged 6–11 months, and 85.5% of children aged 12–23 months [19]. Notably, these DHS data categorize the burying of feces as “safe disposal”, a definition that has been contested by experts in the field [20]. In studies conducted in sub-Saharan Africa, predictors of the safe disposal of child stool included: older mothers [21,22], urban residence [21,22,23,24], wealthier households [22,23,24,25], higher maternal education [22,23], access to an improved toilet facility [23,25], and older children [23,24].

By definition, access to improved sanitation facilities are a prerequisite to the proper disposal of human waste. While there has been a global push towards improving access to effective sanitation facilities and general hygiene, coverage of these basic services remains lacking [26]. In 2015, an estimated 2.3 billion people worldwide lacked basic sanitation facilities—ones that are improved and not shared with other households. Additionally, 892 million people practiced open defecation, the lowest level in the JMP sanitation ladder used to measure progress towards WaSH Sustainable Development Goal (SDG) targets [26]. Compared to other regions, sub-Saharan Africa faired poorest with 28% of its population using basic sanitation facilities, compared to an average of 68% globally [26]. In Tanzania, 19% (36% urban vs. 11% rural) of households have access to improved, non-shared toilet facilities [19]. An estimated 10% of Tanzanians have no access to a sanitation facility and practice open defecation [19]. Geographic inequities in access and availability of basic WaSH services persists, particularly among the urban poor and rural populations [26]. In the rural Geita region for example, only 9.6% of households have access to improved, non-shared toilet facilities [19]. This service gap continues to hamper progress towards the SDG target of attaining universal access to sanitation. 

Public health programs have largely focused on the overarching issue of sanitation, with few interventions specifically addressing the handling and disposal of child feces [17,27,28]. Even in contexts where large scale sanitation projects have been implemented to some success, child feces are still handled improperly [16,27]. Furthermore, research conducted in this area, and within sub-Saharan Africa have focused on identifying predictors of safe/unsafe disposal of child feces, and not the underlying cultural and behavioral factors leading to the practice [21,22,23,24,25]. There remains a gap in knowledge around cultural and behavioral barriers to safe disposal of child feces in rural Tanzania and similar settings across sub-Saharan Africa. Continued neglect of the handling of child feces may undermine larger WaSH goals in sub-Saharan Africa and globally. 

The aim of the larger qualitative research study, in which findings presented here emerged, was to understand the major health adversities experienced by households, and how members of a family address these challenges. Findings on the handling of infant feces emerged incidentally and organically. In this paper, we report on these findings and discuss the perceptions, practices, and changing views of the disposal of infant feces in a rural village in the Geita Region of Tanzania. We also discuss the spectrum of perceptions regarding the purity of infant feces, and how these understandings have evolved over time. The goal of this paper is to highlight a global health issue of improper handling of child feces and propose targeted interventions to address it and improve child health outcomes in the region.

## 2. Materials and Methods

### 2.1. Study Setting

Administratively, Tanzania is divided into regions, districts, divisions, wards, villages, and lastly hamlets. This study was conducted in one rural village and all its 5 hamlets, in the Geita region of Tanzania. The Geita region, located in the North Western Tanzanian Lake zone, is composed of five districts with 1.7 million inhabitants [19]. The region, primarily known for its gold mining industry, was formed in 2012 from parts of neighboring Shinyanga, Mwanza, and Kagera regions [19]. Overwhelmingly, inhabitants of the village in which the work was conducted are small-scale subsistence farmers. The village relies on Lake Victoria for both small-scale and commercial fishing activities. The two main ethnic groups found in the village are the Sukuma and Zinza tribes.

### 2.2. Study Design

This qualitative study was conducted as part of a broader household survey on WaSH and nutrition between June and August 2018. The survey collected household-level: demographic and educational attainment; economic status; access to water and sanitation facilities; dietary diversity; child nutritional status; and women empowerment and status data. The smaller qualitative study sought to contextualize findings from the household survey. Specifically, the qualitative study aimed to understand the major health concerns in the village, household vulnerability to health-related adversity, strategies for addressing health challenges (resilience), decision-making modalities in the home, and the status of women in the village. 

The qualitative study design was informed by principals of grounded theory [29]. Semi-structured focus group discussions (FGDs) were conducted with women, men and leaders in the rural village in Geita region. FDGs with women were used to elicit opinions on their status within their communities, the health adversities experienced, and the strategies used to overcome those challenges. FGDs with men incorporated male perspectives and served to triangulate data collected from women. Specifically, the discussions focused on the decision-making process in the home, aspects they respect about women and how they contribute towards overcoming health adversities. FGDs with community leaders offered a societal backdrop, with which we contextualized data collected from all other respondent groups. Data collection instruments (guides) included a list of broad questions covering the aforementioned topics, with more detailed sub-questions and probes to clarify specific issues. The FGD guides were semi-structured, allowing the facilitators to ask the questions in any order to accommodate the flow of the discussion. 

Of note, questions specific to the management of infant feces were not explicitly posed to our respondents. Rather, this topic organically emerged in the first two FGDs as respondents were discussing health issues experienced in the village (such as diarrheal diseases). Given this incidental finding, the FGD guide was adjusted to include questions on the management of child feces in order to get more nuanced understanding of these practices. All other questions in the FGD guides remained the same. Saturation was achieved when researchers found the same responses at each FGD. Despite reaching saturation, questions on the management of child feces were posed to all FDGs to allow for the triangulation of data across respondent groups. 

### 2.3. Training and Data Collectors

One facilitator, and the Principal Investigator (PI) of the qualitative study, conducted the FGDs. The facilitator was a secondary school teacher, who holds a bachelor’s degree in education. The PI, who is bilingual in English and *Kiswahili*, has graduate-level training in social sciences and public health. Training involved a daylong instructional session and covered modules, including, qualitative data collection methods and research ethics. Testing of the data collection instruments was twofold. First, the PI and facilitator practiced the questions with data collectors of the larger study. This included two community health workers (CHWs) and four data collectors who held teaching certificates. All data collectors on the team were inhabitants of the village. Second, a formal pilot test was conducted in two FGDs consisting of 4 women and 4 men respectively. This was done to ensure that the questions were relevant, flowed well and were understandable to our target group. Data from these tests have not been included in this manuscript. We did not conduct a practice FGD with community leaders, as we were limited by the number of leaders in the village.

### 2.4. Participant Sampling

Participants were included in this study if they were residents of the Geita region (lived in the region for at least 6 months and spend at least 6 months of the year in the region). To ensure diversity, participants were selected purposively for participation in FGDs to represent variety in age, parity, occupation and religion. Given that our study did not explicitly seek to study how participants handle their infant’s feces, having or being a caregiver to children under five years of age was not used as an inclusion criterion into the study.

Following Tanzania’s administrative protocol, researchers introduced themselves to relevant government officials and sought village-level permissions to carry out the study. A community health worker (CHW), embedded in the village, assisted in the identification of potential study participants, based on the inclusion criteria. Research staff then met with each participant to explain the study’s goal and obtain written informed consent. For participants who were unable to write, the consent form was read to them, and a thumbprint was used to indicate their willingness to participate in the study. 

### 2.5. Data Collection Procedures

All FDGs were conducted in a central location of the participants’ choosing. This included a local primary school, a community-owned multipurpose building and the local government building (for FGD conducted with leaders). To encourage unrestricted discussions, FDGs with community members were stratified by sex. Female FGDs were further stratified by age (≤30 years and >30 years) to promote richer and more open dialogue. Based on discussions with local CHWs and leaders, the stratification of male FGDs by age was found to be unnecessary, since it was culturally acceptable for men of all ages to engage in open conversation. Similarly, stratification by sex for discussions with community leaders was deemed unnecessary, as male and female leaders were viewed as having equal power. Furthermore, free and open dialogue in individual FGDs was encouraged through rapport-building prior to the discussions by casual conversations between investigators and participants. During discussions, facilitators reiterated the fact that we were interested to hear their experience, and were not looking for right/wrong answers. We reassured respondents that identifiable responses will not be shared, and that responses would not influence the care they receive. Additionally, the facilitator was trained on inclusive facilitation techniques, such as asking “does anyone else have something to add?”, to ensure active participation by all respondents. 

While in the field, researchers noted that two hamlets were similar in economic status and close in proximity. Due to the businesses and government presence in those hamlets, the area was described as being peri-urban. The remaining three hamlets, on the other hand, were less economically robust as they did not have established business centers or markets. These hamlets were further away from the economic center of the village and were considered more rural. The FGDs were therefore grouped by these two locations: peri-urban and rural. 

### 2.6. Data Analysis 

All FDGs were digitally recorded and transcribed verbatim following data collection. In line with Grounded theory, the researchers used an inductive research and analysis approach, which allowed the data collected to drive the exploration and detection of explanatory theories [29]. Data analysis was initiated in the field with the facilitator and PI debriefing after each FGD in order to identify emerging themes, areas for further exploration and identify saturated topics [30]. To maintain meaning and integrity, review of primary data was conducted in the language in which it was gathered, *Kiswahili*, by bilingual investigators. Researchers thematically analyzed the transcripts and identified recurring themes and patterns in the dataset using the qualitative management software, Atlas.ti (Scientific Software Development Gmbbh, Berlin, Germany) [31]. Emerging themes were openly coded by the lead investigator, interpreted collaboratively by all investigators, and summarized with investigators native to the region to ensure accurate interpretation. Data was triangulated across the three respondent groups, with comparisons on the findings made by study investigators. Study findings are reported according to the standards for reporting qualitative research (SRQR) (Supplementary Table S1) [32]. 

### 2.7. Ethical Considerations 

Ethical approval was received from the Tanzanian National Institute for Medical Research (NIMR) and the University of Arizona ethical boards. All participants consented prior to enrollment. 

## 3. Results 

### 3.1. Participant Characteristics 

Seven FGDs, each with 8 participants (56 total participants), were held (Table 1). The discussions included younger women aged 30 and below (*n* = 2 FGDs; 16 participants), older women over 30 years old (*n* = 2 FGDs; 16 participants), men (*n* = 2 FGDs; 16 participants) and leaders (*n* = 1 FGD; eight participants) from both the peri-urban (*n* = 8 FGDs) and rural (*n* = 8 FGDs) hamlets. No participants declined participation in the study, and all consenting individuals engaged in their respective FDGs from start to finish. That is, there was no participant dropout. Discussions lasted for an average of 108 min and ranged from 88 to 126 min. 

On average, the women participating in the FGDs were younger (mean age = 32.8; range 18–66) compared to the men (mean age = 46.4; range 23–77), and the leaders (mean age = 50.1; range 22–67). Generally, educational attainment was low in the population. Men were the least educated, with 18.8% not completing primary school, compared to 12.6% of younger women, 6.3% of older women and none of the leaders. Majority of the older women, men and leaders participating in the FGDs were married. However, about half of the younger women were single. On average, younger women had the fewest children (mean 1.5; range 0–4), compared to older women (mean 5.4; range 1–10) and men (mean 6.5; range 0–20).

The management of child feces emerged unprompted in the FGDs with older women and men from rural sites, which were conducted first. In the remaining FDGs, personal experiences, opinions, and insight on the disposal of stool from children were solicited. Major themes emerging from the dataset are summarized in Table 2. These themes are expounded upon in the sections that follow.

### 3.2. Access to Improved Water and Sanitation Facilities, Water-Related Illnesses and Infant Feces

When discussing health challenges experienced in their village, water-related illnesses emerged as a recurring theme across all FGDs. Respondents described these afflictions most commonly as ‘diseases of the stomach’ (*magongwa ya tumbo*), which included diarrhea, helminths and fevers (Table 2). Both young women and community leaders noted that typhoid was a health challenge in the village. The issue of bloody diarrhea emerged as a health challenge in the community leader and men FGDs, alluding to dysentery as a health concern in the village. Community leaders were the only group to also discuss schistosomiasis (*kichocho*) and infection by amoebas (referring to the organism using the English term) as prominent water-related diseases in the village. One community leader explains:

“You find in schools that many children use just a few toilets. They go into those toilets without any shoes—without slippers, without anything. That child is not safe—you will find that the child has *kichocho*. They will start to have a big stomach, and people think the child is healthy. Only to find out that it is those parasites that are doing their job.”—FDG with community leaders, peri urban location

When asked to reflect on the causes of these *magongwa ya tumbo*, respondents in all FGDs explained they were a result of consumption of unsafe water, lack of unimproved latrines, and open defecation. An older woman residing in a rural site lamented, “If you see the water we drink from the spring, you will have pity on us.” Participants across all respondent groups reported that they generally do not have access to clean water, sourcing their drinking water from naturally occurring unprotected springs. Furthermore, respondents reported that drinking water fetched from these unimproved sources was solemnly treated or boiled, thereby leading to water-related illnesses. Open defecation and lack of access to improved sanitation facilities were also discussed as contributing to water-related illnesses experienced in the village. Across all respondent groups, a clear link was made between drinking contaminated water and poor disposal of feces, and water-related illnesses. A young woman from a rural site explained how feces can lead to stomach problems by saying:

“Someone can go and use the toilet. They use leaves to wipe themselves. In this act of cleaning themselves, the feces gets on their hands. Later when they get home, they go and cook potatoes. They take those potatoes and eat them without washing their hands. Day after day, they will be disturbed by their stomach.”—FDG with young women, rural location

Leaders, too, expressed concern about the lack of adequate sanitation and safe water, its connection to water-related illnesses, and the role they play in addressing these diseases.

“As leaders, we have not enforced the policy that encourages each household to dig their own latrine in order to stop people from defecating all over the place. This open defecation causes them and people around them to eat their feces that have particles of amoeba, schistosomiasis and other diseases. Therefore, we have to strengthen our laws so that each house has their own latrine. We know that our springs are low—I mean they are down in the valley. So, when it rains, all the feces that are all over the place gets swept into our wells. That is the same water we keep in our containers [at home]. So, if we enforce that it is the responsibility of each household to dig their own latrine, I think defecating all over will stop.”—FDG with community leaders, peri urban location

**Figure 1:**
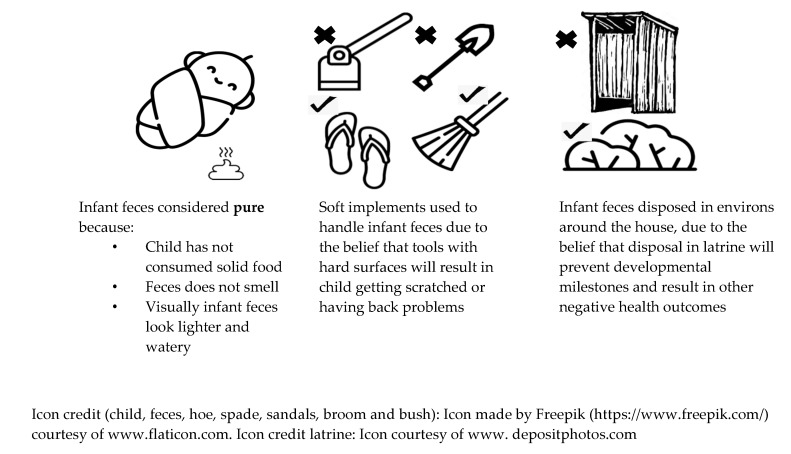
Summary of perceptions, practices and changing perspectives of infant feces in Geita region, Tanzania.

In addition to unimproved and unsafe water sources used by majority of the village inhabitants, participants identified traditional *Kisukuma* views on infant feces, children under the age of 1, as a contributing factor to the high levels of water-related diseases, one participant noting, “this is why fevers [in our village] never end” (FGD with young women, peri-urban location). Developing unprompted and organically, respondents explained the view of infant feces being pure, and requiring specific handling and disposal (Figure 1).

### 3.3. Perceptions of Infant Feces as Pure 

Participants across all respondent groups discussed the traditional belief, still held by many in the community, that infant feces are innocuous. The primary reason for this view was the fact that the infant has yet to consume any solid foods, which would hold contaminants within it. Therefore, since the child relies only on breastmilk, viewed as pure, their feces too, would remain benign. As one participant explained:

“The feces of young children were traditionally seen as harmless because from the time a child is born to about when they are six months [old], they have not eaten any [solid] food. Just the milk of their mother. So, their stool has no smell or bacteria. That is why you do not need to throw it in the toilet. Handling that stool you will not even give you a rash”—FGD with older women, peri-urban location

However, as the child matures and transitions to consuming solid foods, their feces cease to be considered pure. This transition from pure to impure feces is captured by a participant, who explains:

“What I know is that if a child is young, between birth and six months, their feces is clean. Not a child over six months. Over the age of six months, that child has started to eat regular food. Their feces are not comparable to a child who has not eaten regular food. What I know and believe is that a child who is only breastfeeding has good stool.”—FGD with men, rural location

The lack of an offensive smell was also viewed as a factor lending credence to the purity of an infant’s excreta. One participant, recounting from personal experience said:

“Even when you are in bed with your wife and [infant] child and they defecate on the sheets, you just keep quiet. The feces do not smell. Your wife will just wipe the baby and you go back to sleep. It is not like an older child. If a five-year-old child does the same—a child who has eaten ugali [cornmeal dish], you will chase them out, ‘get out of here’”—FGD with men, rural location

Furthermore, visual distinctions in consistency and color between infant feces and the excreta of older children and adults, was discussed as a reason for the difference in handling the fecal matter. A woman in an FGD explained:

“For young children, there are different kinds of feces. There is the light one and the solid one. You will find that some women wipe the light feces using a cloth that they reuse for a week. When they wash the piece of cloth, they pour the water in the *“zizi la ng’ombe*” (where cattle are kept).”—FGD with older women, peri-urban location

### 3.4. Children Defecation Sites and Fecal Disposal Practices 

Respondents noted that the floor was the primary defecation site for ambulatory children under five years of age in the village, with cloth diapers being used occasionally. Infants, on the other hand defecated on pieces of fabric, cloth diapers, or on the floor assisted by a caregiver. As a result of the view that infant feces are harmless, respondents reported disposal of the excreta in places other than the toilet. As one participant noted, “You will find some people who say, “does a child’s feces really smell?” they just throw it anywhere.” (FGD with young women, peri-urban location). In another FDG with men, a participant explained, “A lot of women have this practice—if a young child under the age of 6 months defecates here, they will take it and throw it off to the side, not in the latrine” (FGD with men, rural location).

### 3.5. Perceived Consequences of “Improper” Disposal of Infant Feces

The location of fecal disposal, and the manner in which the infant feces is handled emerged as an important consideration for caregivers. If an infant, particularly ones who are under six months and still exclusively breastfeeding, defecates on the ground, participants explained that the feces should not be handled with any implement with hard or metal surfaces. This includes hoes, spades and any other farm equipment. Instead, it should be moved using something with a soft surface, including leaves, slippers, brooms made from grass, or a piece of cloth. A young mother recounted her experience, and said, “Like me, I have a child at home, when they go to the toilet [defecate] my mother says, ‘don’t use a hoe [to pick it up]’”—FGD with young women, rural location. 

Participants explained that using tools with hard surfaces to handle an infant’s feces could have immediate and future health implications for the child. As one participant explained:

“People say that if you use a hoe or spade to pick up the feces of a very young child, that it will scratch the back of the child. That is why they use sand to cover it, and use a slipper to move it to the hoe—you do not scoop it up with the hoe”—FGD with young women, peri-urban location

In a different FGD, a participant noted:

“They used to say that [if you use a hoe or spade to pick up their feces], when they grow up, the child will have back problems. That their back will feel like it is being scraped in the same way that a hoe was used to scrape the child’s feces.”—FGD with men, peri-urban location

In the same FGD, another participant explained a different consequence related to the handling of infant feces by noting:

“They also used to say that [if you use a hoe or spade to pick up their feces], the teeth from their top gum will come in first. In Kisukuma, we say that if your top teeth come in first, you will die when you are young before you become a man”—FGD with men, peri-urban location

In addition to the perceived negative consequences associated with using hard-surfaced tools to handle infant feces, disposal of the fecal matter into a latrine was viewed as having negative repercussions for the infant. The consequences centered on the child’s development, with disposal of an infant’s feces in a latrine thought to impede their achieving developmental milestones. One participant noted they have heard that, “If you throw the feces of a child who does not have teeth yet, it is a mistake. They will not get teeth” FGD with young women, rural location. In another FGD, a participant discussed the link between feces disposal and child milestones, by saying:

“Traditionally, we did not use hoes [to pick up an infant’s feces] and did not throw [the feces] in the pit latrine. If you threw their feces in the latrine, people said that the child will not walk on time, they will be deaf.” The participant joked “Is that why we have so many deaf people now? [Laughter]”—FGD with older women, peri-urban location

In explaining the rationale behind the perceived consequences of “improper” fecal disposal, one participant explained, “If you throw the feces in the toilet, it is like you are throwing the child in the latrine” (FDG with men, rural location). 

### 3.6. Changing Views on the Handling of Infant Feces 

Despite the pervasive tradition on handling of infant feces, participants were clear that this view is not held by the entire community. Following lengthy discussions on the subject, one participant offered this point of clarification:

“Let us clarify, that this belief [that infant feces is pure and should not be disposed of in a latrine] is not held by everyone. The way we are talking, it makes it seem like we all hold that belief. In my house, feces are feces, even that of a young child. It is treated the same… It was an old belief, but things are changing now.”—FGD with men, rural location

One participant exemplified this change in practice by recounting how his views have changed over time:

“A long time ago when I first got a wife and we had a child, I saw that my wife did not use a hoe or a spade [to pick up] the feces of a young child, and it surprised me. She used the leaves of a tree or a heavy cloth to carry the dirt away and throw it in the bushes. However, after education, we realized that that is an old belief from a long time ago. It is not something that make sense and something that we should not follow. If the child does not have any illness, it is ok [to throw their feces in the latrine]. After we learned this, that practice has ended in many households. In my family we do not believe that anymore”—FGD with men, peri-urban location

While many FDG participants noted that they do not currently believe in this tradition, they mentioned that not everyone in the community has abandoned the belief. “Now people throw [infant feces] in the toilet, but not everyone. There are still people who hold this faith. Those who are a little educated know that [infant feces] is dirty” (FGD with older women, peri-urban location). 

As primary caregivers, women were viewed as the primary group that propagates and performs this practice.

“This belief is mostly with the women, when they are together they discuss these things and spread it among themselves… a lot of women have this practice—if a young child under the age of six months defecates here, they will take it and throw it off to the side, not in the latrine”—FGD with men, rural location

## 4. Discussion

This present study sought to understand vulnerability to, and resilience towards health challenges faced by families in a rural village of the Geita region of Tanzania. Data were gathered through FGDs with men, women, and leaders from that community. Other than lack of sanitation facilities and safe water, local perceptions and practices embedded in traditional practices around the handling and disposal of infant feces emerged organically from the discussions as a custom contributing to recurrent water and sanitation related diseases in the village. Respondents noted that the feces of infants, who have yet to consume solid foods, are viewed as being harmless. The feces are therefore disposed of unsafely in the environments surrounding the home, which under the JMP definition constitutes open defecation [18]. Moreover, the safe disposal of stool of an infant under the age of six months—in a latrine—was described as impeding the development and adversely affecting the health of the child. Despite still being practiced, our respondents described these views and practices as outdated and changing.

Results from our work corroborate the low rates of safe child feces disposal documented in the Tanzanian DHS (66%), and contributes to understanding these persistently low rates in the region [19]. Unlike other work where communities expressed poor cognitive association between feces and disease, our respondents recognized this link [33]. Despite this acknowledgement, the feces of infants younger than six months were still reportedly disposed of unsafely. Previous work in Ethiopia, Nigeria, and other developing countries also showed unsafe disposal of child feces, which may be attributed to the belief that they are harmless [16,21,23,24,25]. 

Work conducted in urban Peruvian sites showed that feces were considered increasingly impure with age—that is, adult feces thought of as being dirtier than that of children—because of the amount of food consumed by adults [33]. Even among children, a hierarchy of fecal purity emerged, with infants seen as having the least dirty stool given they were yet to consume solid foods [33]. The perception of feces, therefore, was shown to evolve based on infant breastfeeding and mixed feeding, where the purity of infant feces is compromised once the infant is weaned [33]. In the Tanzanian context, the vast majority of children are breastfed in their lifetime (98%), and more than half (59%) exclusively in the first six months of life [19]. Therefore, understanding the intersection of weaning and fecal purity of a child is important. Further still, the changes in smell of the child’s stool during the transition between exclusive breastfeeding and complementary feeding was viewed as an indicator of impurity in our work as well as that of other scholars [11,33]. Changes in stool appearance, consistency, and smell, used as markers of purity/impurity of feces in our study, could be explained by the transformation of a child’s microbiome over time. Research on the digestive microflora suggests the bacterial profile of healthy infants changes with age, and as children consume a diet similar to that of adults [34].

The location and socioeconomic status of our research participants—a low income rural village—is also in line with other research that has shown these to be determinants of unsafe child feces disposal [23]. Additional documented determinants of unsafe child feces disposal include the lack of improved toilet facilities [21,23]. However, even when latrines are available, concern for the safety of older ambulatory children has been cited as an issue of concern for having children use latrines, fearing that they may fall in since the design is not optimal for use by children [11]. Safe disposal of the stool of children is limited by the availability of, and access to improved sanitation facilities. In the context of our work, respondents identified this lack as a health challenge in their village. More broadly, the Tanzanian DHS reports greater levels of safe disposal of child feces in households with improved toilets (90% and 82% in households with shared and non-shared facilities respectively), compared to those with unimproved facilities (67%) [19]. 

Our work showed potential sources of fecal contamination and exposure resulting from child defecation sites, implements used to handle stool and sites for excreta disposal. In addition to the act of disposing infant feces, alternative ways in which fecal matter may enter the environment should be considered as points of intervention. These pathways, documented by Majorin and colleagues (2017), include the surface on which the child defecates, the tools used to handle the feces, anal cleaning and handwashing, feces disposal and the sanitation system [35]. Other work has documented remnants of fecal matter on the infant’s or caregiver’s clothes as additional sources of exposure [33]. 

The changing views of safe disposal of infant feces in our study site suggests a foundation on which public health interventions can be developed. These interventions can be informed by others who suggest an intervention should address local conceptualizations—in this case, conceptions of purity of infant feces and patterns of home care behaviors [36]. As primary caregivers in this context, mother-focused interventions would provide an avenue for maximum effect. However, studies have shown that older children also share in the responsibilities of rearing their younger siblings [37]. Therefore, in developing interventions, it is important to understand the ideas and practices of older children in relation to the disposal of feces of those they take care of. Furthermore, these older children should be included in school- and community-based educational programs on the safe disposal of child feces. Of note, the apprehension held by caregivers on the safe disposal of their infant’s feces should be taken into consideration in developing culturally relevant and economically viable interventions. Such interventions would address and allay concerns about the health and wellbeing of infants. Other research has highlighted that the use of latrines, as promoted by many sanitation projects may not be useful for disposal of ambulatory child feces because they are not viewed as appropriate for young children [11]. Low-cost products such as reusable diapers, potties, and latrine seats have been suggested as interventions to enable the safe disposal of child feces, and can be applied in our research setting [38]. 

This study was limited by challenges associated with qualitative methods, specifically social desirability bias. Given the benign nature of subjects under investigation, it is unlikely this bias altered respondent responses. However, to mitigate this limitation, study investigators invested in rapport-building activities with the respondents, as part of the larger community, over the course of six weeks prior to holding the FGDs. Additionally, due to the design of the study, we were unable to quantify the extent to which the perception of infant feces as innocuous is pervasive in this village, and the number of households that practice this behavior. Future studies would benefit from a mixed method design, including participant observations, that allows for this quantification. Additionally, the recruitment of participants with children under the age of six months would garner direct experience with individuals actively handling infant feces. Lastly, the fact that this study was conducted in one village largely inhabited by two ethnic groups (the Sukuma and Zinza) may limit the generalizability of the results to other regions with different cultural and ethnic groups. However, documentation of infant feces handling and practices in this population remains important, and is reflective of other research in similar settings.

Strengths of this study include its contribution to the understanding of the handling and disposal of infant feces in low resource settings. Despite the importance of the subject matter, very few Tanzania-specific studies are available. Furthermore, to our knowledge, none of the studies focus solely on the practices around the handling of feces of infants. Our study also triangulated findings across a diverse sample, in terms of age, gender, and social status.

## 5. Conclusions

The safe disposal of child feces remains a formidable yet neglected barrier to fully actualizing WaSH targets. Improper handling and disposal practices continue to contribute toward morbidity and mortality across all age groups, with children under the age of five bearing the brunt of water and sanitation related illnesses. Understanding practices and perceptions of child feces can assist in developing WaSH interventions to improve child survival and improve health outcomes. Our findings suggest that while unsafe disposal of child feces remains a challenge, changing views on the practice offer an opportunity for public health intervention. Based on our findings, there is a need to integrate culturally relevant educational interventions into current WaSH interventions, inclusive of caregivers—both mothers and older children who often care for their younger siblings.

## Figures and Tables

**Table 1:** Summary of respondent characteristics engaged in focus group discussions in Geita region Tanzania, by respondent group.

Characteristic	Young Women (*n* = 2 FGDs; 16 Participants)	Older Women (*n* = 2 FGDs; 16 Participants)	Men (*n* = 2 FGDs; 16 Participants)	Leaders (*n* = 1 FGD; 8 Participants)
**Age;** mean, (SD), Range	22.3 (3.3) 18–29	43.3 (9.6) 30–66	46.4 (15.4) 23–77	50.1 (14.0) 22–67
**Location;***n* (%)—participants				
“Peri-urban” hamlet	8 (50.0)	8 (50.0)	8 (50.0)	5 (62.5)
Rural hamlet	8 (50.0)	8 (50.0)	8 (50.0)	3 (37.5)
**Educational attainment;***n* (%)—participants				
No formal education	1 (6.3)	1 (6.3)	1 (6.3)	0 (0.0)
Some primary schooling	1 (6.3)	0 (0.0)	2 (12.5)	0 (0.0)
Completed primary school	9 (56.3)	13 (81.3)	11 (68.8)	6 (75.0)
Some secondary schooling	0 (0.0)	1 (6.3)	0 (0.0)	0 (0.0)
Completed “O” levels	4 (25.0)	0 (0.0)	1 (6.3)	1 (12.5)
Completed “A” levels	1 (6.3)	0 (0.0)	0 (0.0)	1 (12.5)
Higher education	0 (0.0)	1 (6.3)	1 (6.3)	0 (0.0)
**Marital status;***n* (%)—participants				
Single/never married	9 (56.3)	1 (6.3)	1 (6.3)	0 (0.0)
Married	5 (31.3)	12 (75.0)	15 (93.8)	8 (100.0)
Divorced/separated	1 (6.3)	2 (12.5)	0 (0.0)	0 (0.0)
Widow/Widower	1 (6.3)	1 (6.3)	0 (0.0)	0 (0.0)
**Number of children;** mean, (SD), Range	1.5 (1.3) 0–4	5.4 (2.2) 1–10	6.5 (5.6) 0–20	--

-- indicates that data on this participant characteristic was not collected.

**Table 2:** Summary of themes emerging from focus group discussions in Geita region Tanzania, by respondent group.

Theme	* Respondent Group			
	Young Women	Older Women	Men	Community Leaders
Access to, and use of improved water and sanitation facilities				
Lack of improved water sources	X	X	X	X
Consumption of contaminated water	X	X	X	X
Lack of improved sanitation facilities	X	X	X	X
Water-related illness experienced in the village				
Diarrhea (*kuhara*)	X	X	X	X
Bloody diarrhea (*kuhara damu)*			X	X
Fever (*homa*)	X	X	X	X
Helminths (*minyoo*)	X		X	X
Typhoid	X			X
Schistosomiasis (*kichocho*)				X
Amoeba				X
Defecation sites for children under the age of five years				
Floor	X	X	X	X
Fabric or cloth diapers	X	X	X	X
Perception that feces from children under the age of six months as innocuous				
Heard	X	X	X	X
Practiced in the past	X	X	X	X
Currently practicing			X	
Issue in the village	X	X	X	X
Reasoning for view of “harmless” feces of children under that age of six months				
Child has not yet consumed solid food	X	X	X	X
Does not smell	X		X	
Visually different from feces of older children		X	X	
Handling of stool from children under the age of 6 months				
Hard-surfaced implements should not be used	X	X	X	X
Soft-surfaced implements should be used	X	X	X	X
Perceived consequences of “improper” handling and/or disposal of infant feces				
Scratched back/back problems	X	X	X	X
Delayed/”incorrect” order of sprouting of teeth	X	X	X	X
Inability to walk	X	X		
Deafness	X			
Generally negative child outcomes	X	X	X	X
Transition of stool from innocuous to harmful begins complementary feeding	X	X	X	X
Changing perspectives on the perception of infant feces from harmless to harmful	X	X	X	X

* Includes respondents from both peri-urban and rural sites. Responses are consolidated as they did not differ by place of residence.

## Supplementary Materials

The following are available online at https://www.mdpi.com/1660-4601/17/9/3084/s1, Table S1: Checklist for Standards for Reporting Qualitative Research (SRQR) for research study on the handling of child feces.
